# Risk factors for and characteristics of community‐ and hospital‐acquired drug‐induced acute kidney injuries

**DOI:** 10.1111/fcp.12758

**Published:** 2022-01-25

**Authors:** Amayelle Rey, Valérie Gras‐Champel, Gabriel Choukroun, Kamel Masmoudi, Sophie Liabeuf

**Affiliations:** ^1^ Division of Clinical Pharmacology Amiens University Hospital Amiens France; ^2^ MP3CV Laboratory, EA7517 Jules Verne University of Picardie Amiens France; ^3^ Division of Nephrology Amiens University Hospital Amiens France

**Keywords:** acute kidney injury, community‐ and hospital‐acquired, drug‐induced, pharmacovigilance, PMSI, risk factors

## Abstract

Drugs constitute one of the leading causes of acute kidney injuries (AKIs) and can appear in community (CA‐AKI) or hospital (HA‐AKI) population. The objectives of the present study of a cohort of hospitalized patients with AKI were to describe the characteristics of drug‐induced AKIs and the patients' short‐term outcomes and assess risk factors for drug‐induced AKIs overall, CA‐AKIs, and HA‐AKIs. Based on a cohort of 1557 hospitalized patients suffering from AKIs based on PMSI extraction and chart review (IRA‐PMSI), drug‐induced AKIs were identified by applying the Naranjo adverse drug reaction (ADR) probability scale. Multivariate logistic regression was used to identify factors associated with CA‐AKIs and/or HA‐AKIs. When considering the 1557 patients who experienced an AKI, 445 (28.6%) of the injuries were drug‐induced (180 CA‐AKIs (40.4%) and 265 HA‐AKIs (59.6%)). Antibiotics, diuretics, and contrast agents were significantly more likely to be involved in HA‐AKIs, whereas antineoplastic, lipid‐lowering drugs, antidiabetics, and immunosuppressive were significantly more likely to be involved in CA‐AKIs. Female sex (odds ratio [OR] [95%CI] = 1.3 [1.04–1.67]), chronic kidney disease (CKD) (OR = 1.8 [1.40–2.67]), and a history of ADRs of any type (OR = 1.3 [1.05–1.73]) were significant risk factors for drug‐induced AKIs. CKD was a risk factor for both CA‐AKI and HA‐AKI. In view of the long‐term impact of AKI on the kidneys and the differences between our CA‐AKI and HA‐AKI subgroups, our present results are interesting for optimizing treatments, limiting the occurrence of CA‐ and HA‐AKIs and (ultimately) reducing healthcare costs.

**List of Abbreviations**
ADR: adverse drug reaction
AKI: acute kidney injury
ATC: anatomical therapeutic chemical
BMI: body mass index a history of hypertension
CA: community‐acquired
CI: confidence interval
CKD: chronic kidney disease
CVD: cardiovascular disease
eGFR: estimated glomerular filtration rate
EMRs: electronic medical records
ESRD: end‐stage renal disease
HA: hospital‐acquired
ICUs: intensive care units
KDIGO: Kidney Disease: Improving Global Outcomes
NSAIDs: non‐steroidal anti‐inflammatory drugs
OR: odds ratio
PMSI: *Programme de médicalisation des systèmes d'information* (French national hospital discharge summary database)
SD: standard deviation

## 1 INTRODUCTION

Acute kidney injury (AKI) is a common, treatable, reversible syndrome that can be immediately life‐threatening and can have serious long‐term consequences [1]. The main impact is on the kidney itself: the development of chronic kidney disease (CKD), accelerated progression to end‐stage renal disease (ESRD), and failure to recover kidney function [2, 3]. The risk factors for AKI have been well studied; they include older age, female sex, high body mass index (BMI), a history of hypertension, cardiovascular disease (CVD), diabetes, CKD, and previous AKI [4, 5, 6]. Based on a cohort of hospitalized patients suffering from AKIs based on PMSI extraction and chart review (IRA‐PMSI), we have recently described different characteristics between hospital‐acquired AKI (henceforth referred to as HA‐AKI) and community‐acquired AKI (henceforth referred to as CA‐AKI). Drug‐induced AKIs accounted for 28.6% of the AKIs [7]. Indeed, the main avoidable risk factor is the use of certain medications [8, 9].

Medications constitute one of the leading causes of AKI [10] and the third to fifth leading cause of hospital‐acquired AKI [11]. According to the meta‐analysis by Cartin‐Ceba et al, each additional nephrotoxic drug increases the risk of developing AKI by 53% [5]. Jacobs et al.'s systematic review showed that iatrogenic AKIs now account for 55–75% of the patients with AKI admitted to intensive care units (ICUs) [12]. Several studies have assessed the role of nephrotoxic drugs, including antibiotics (particularly aminoglycosides, amphotericin, and glycopeptides), immunosuppressive drugs (such as cyclosporine), renin‐angiotensin drugs, contrast agents, non‐steroidal anti‐inflammatory drugs (NSAIDs), and diuretics [10, 13, 14, 15, 16]. Various physiopathological mechanisms are involved in drug nephrotoxicity: impaired glomerular filtration, tubular cell toxicity, interstitial nephritis, crystal nephropathy [15, 16]. A single drug class may even be responsible for several types of damage [17]. Risk factors for drug‐induced AKI described in the literature include a history of diabetes, hypertension [18], and adverse drugs reactions (ADRs) [19]. Drug‐induced AKI has been particularly studied in ICUs, due to the number of drugs administered to patients in this medical unit (twice as much as in other departments) and the nephrotoxicity of many of these drugs [11, 20].

Drug‐induced HA‐AKI is a common problem; in a cross‐sectional survey conducted in mainland China, it accounted for 37.5% of all hospital‐acquired AKIs [21]. Robert et al. reported that drug‐induced CA‐AKI accounted for 58.8% of all CA‐AKIs [8]. Although CA‐AKI accounts for a non‐negligible proportion of hospital admissions, it has been less extensively studied than HA‐AKI. To the best of our knowledge, few studies have compared drug‐induced community‐ and hospital‐acquired AKI.

The objectives of the present study of a cohort of hospitalized patients with AKI were to describe the characteristics of drug‐induced AKIs and the patients' short‐term outcomes and assess risk factors for drug‐induced AKIs overall, CA‐AKIs, and HA‐AKIs.

## 2 MATERIALS AND METHODS

This study was conducted in a university hospital (Amiens Picardie University Hospital). In 2019, this hospital had 1664 beds and places, including 1238 in medicine, surgery, and obstetrics wards. A total of 60 134 patients were admitted during this year, including 289 to the transplantation unit (82 for kidney transplantation), 2566 to the cardiothoracic surgery unit, 787 to the ICUs, and 1285 to the nephrology department.

### 2.1 Data source: Cohort of hospitalized patients with drug‐induced AKIs

A single‐center retrospective study (“IRA‐PMSI”) from January 1, 2019, to June 30, 2019, was performed to build a cohort of hospitalized patients suffering from AKIs based on PMSI extraction and chart review. The IRA‐PMSI study's objectives and procedures were approved by an independent ethics committee (CPP Nord Ouest II, Amiens, France; reference: RCB 2020‐A00556‐33), and the study was registered at ClinicalTrials.gov (NCT04923750). The primary objective of the IRA‐PMSI study was to assess the ability of several ICD‐10 codes to identify AKIs. Results have already been published, such as description of the patient's selection which we described in the Data S1 [7]. The present report describes one of the IRA‐PMSI study's secondary objectives: to describe the characteristics of drug‐induced AKIs and to assess their risk factors.

### 2.2 Definition of drug‐induced AKI

In order to determine whether a given AKI identified with our algorithm (Figure S1) was drug‐induced or not, a pharmacist with expertise in pharmacovigilance and pharmacology (employed at the Amiens Regional Pharmacovigilance Centre) applied the Naranjo ADR probability scale to each drug delivered in hospital in the 48 h before the AKI [22]. This validated tool is mainly used to probe the causal nature of relationships between drug and adverse event through 10 questions on the event's chronology, symptom profile, and literature data [23]. After calculation of the total Naranjo score, each drug was assigned to one of the following probability categories: definite ≥ 9, probable 5–8, possible 1–4, and doubtful ≤0. The pharmacovigilance expert considered that the drug was imputed for AKI that if the score category was “possible,” “probable,” or “definite” (i.e., any score other than 0). For 100 randomly selected drug prescriptions, another pharmacovigilance expert applied the Naranjo scale; the correlation between the two expert's scores was good. The candidate drugs' Anatomical Therapeutic Chemical (ATC) classes were recorded. By applying our algorithm (Figure S1), drug‐induced AKIs were classified as CA‐AKIs or HA‐AKIs.

### 2.3 Clinical and demographic information

For each identified AKI, we collected several clinical and demographic information from the patients' EMRs. The demographic variables included age, sex, and BMI. The comorbidities considered as risk factors for AKI were hypertension, diabetes, dyslipidaemia, heart disease, cancer, gout, CKD (stage ≤3A, [estimated glomerular filtration rate (eGFR) ≥ 45 mL/min/1.73 m^2^], 3B [eGFR between 30 and 44 mL/min/1.73 m^2^], 4 [eGFR between 15 and 29 mL/min/1.73 m^2^], or 5 and not on dialysis [eGFR <15 mL/min/1.73 m^2^]), nephrectomy, kidney transplant, previous AKI, and a history of ADRs. Lastly, clinical data related to the episode of AKI were gathered: the medical unit, KDIGO grade, length of hospital stay, use of dialysis or not, and outcome.

### 2.4 Statistics

Descriptive statistics were used to characterize the study population. Patients were divided according to whether the AKI was drug‐induced or not and then whether the injury was acquired in the community or in hospital. Groups were compared using a chi‐squared test or Fisher's test, depending on the data distribution.

The prescription drugs administered to patients with CA‐AKI versus HA‐AKI were assessed using a chi‐squared test or Fisher's test.

To compare drug‐induced AKIs with non‐drug‐induced AKIs, we calculated the crude odds ratio (OR) [95% confidence interval (CI)] and the *P* value. Next, we used multivariate logistic regression to model the relationship between risk factors and drug‐induced AKI, by taking the non‐drug‐induced AKI group as a reference. The potential adjustment variables were age, sex, obesity (defined as a BMI ≥ 30 kg/m^2^), and risk factors for AKI (i.e., hypertension, diabetes, dyslipidaemia, heart disease, cancer, gout, CKD, nephrectomy, kidney transplant, previous AKI, and a history of ADRs). To optimize the model's fit, we used a parsimonious model that included all variables with *P* < 0.2 in a univariate analysis or contextual interest.

In a secondary analysis, the above‐mentioned values were calculated for the CA‐AKI and HA‐AKI subgroups (relative to the non‐drug‐induced AKI group). Likewise, same variables were used to determine the crude OR [95%CI] (i.e., age, sex, obesity, and risk factors for AKI). Lastly, the multivariate logistic regression model included all the variables with *P* < 0.2 in a univariate analysis or contextual interest.

The threshold for statistical significance was set to *P* < 0.05. All analyses were performed using R software (version 3.6.0, R Foundation for Statistical Computing, Vienna, Austria) [24].

## 3 RESULTS

### 3.1 Characteristics of the study population

**FIGURE 1:**
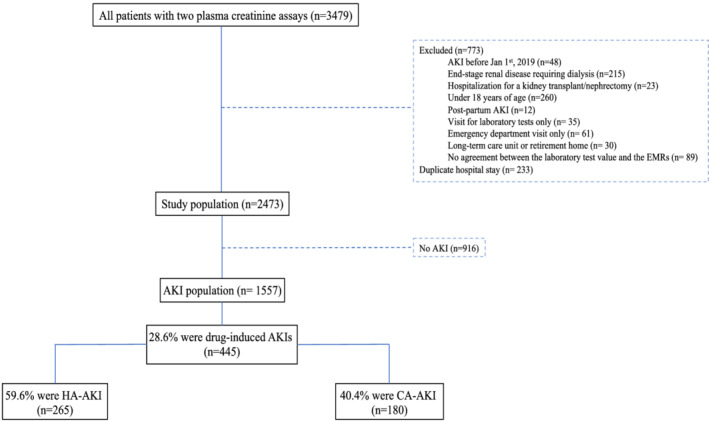
Study flow diagram. AKI, acute kidney injury; CA, community acquired; EMR, electronic medical record; HA, hospital‐acquired

Between January 1, 2019, and June 30, 2019, 3479 of the patients admitted to Amiens‐Picardie University Hospital had two creatinine measurements (Figure 1); 2473 patients (773 with exclusion criterion and 233 with duplicate hospital stay) represented the study population included; 1557 of the 2473 patients had experienced an AKI. Most episodes of AKI were identified from both the EMR review and the laboratory results (54.8%); a lower proportion (33.1%) were only physician‐diagnosed (Table S1). In the AKI population, the mean age (standard deviation (SD)) was 71.3 (15.1), and 45.7% were women (Table 1).

**TABLE 1:** Baseline characteristics of study participants, overall and according to the type of AKI (drug‐induced or non‐drug‐induced)

	All patients with AKI	Drug‐induced AKI	Non‐drug‐induced AKI	*P* value^a^
	1557	445 (28.6)	1112 (71.4)	
**Demographics**				
Age, y, mean (SD)	71.3 (15.1)	72.1 (14.6)	71.0 (15.3)	0.23
**Women, *n* (%)**	**712 (45.7)**	**227 (51.0)**	**485 (43.6)**	**<0.001** *
**BMI, kg/m** ^**2**^ **, mean (SD)**	**27.7 (6.7)**	**28.8 (6.9)**	**27.3 (6.6)**	**<0.001** *
**Obesity (BMI ≥30 kg/m** ^**2**^ **), *n* (%)**	**461 (29.6)**	**153 (34.4)**	**308 (27.7)**	**<0.001** *
**Comorbidities** ^b^				
**CKD, *n* (%)**	**445 (28.6)**	**180 (40.4)**	**265 (23.8)**	**<0.001** *
**CKD stage**				**<0.001** *
**≤3A (≥45 mL/min/1.73 m** ^**2**^ **)**	**215 (13.8)**	**79 (17.8)**	**136 (12.2)**	
**3B (30–44 mL/min/1.73 m** ^**2**^ **)**	**148 (9.5)**	**66 (14.8)**	**82 (7.4)**	
**4 (15–29 mL/min/1.73 m** ^**2**^ **)**	**73 (4.7)**	**33 (7.4)**	**40 (3.6)**	
**5 (<15 mL/min/1.73 m** ^**2**^ **) (not on dialysis)**	**9 (0.6)**	**2 (0.4)**	**7 (0.6)**	
**Hypertension, *n* (%)**	**1092 (70.1)**	**340 (76.4)**	**752 (67.6)**	**<0.001** *
**Diabetes, *n* (%)**	**544 (35.0)**	**176 (39.6)**	**368 (33.1)**	**0.016** *
**Dyslipidaemia**	**569 (36.5)**	**194 (43.6)**	**375 (33.7)**	**<0.001** *
**History of CVD, *n* (%)**	**757 (48.6)**	**250 (56.2)**	**507 (45.6)**	**<0.001** *
Gout, *n* (%)	112 (7.2)	39 (8.8)	73 (6.6)	0.13
Cancer, *n* (%)	456 (29.3)	136 (30.6)	320 (28.8)	0.48
History of kidney transplant, *n* (%)	49 (3.1)	20 (4.5)	29 (2.6)	0.05
History of nephrectomy, *n* (%)	91 (5.8)	31 (7.0)	60 (5.4)	0.23
**History of AKI, *n* (%)**	**242 (15.5)**	**93 (20.9)**	**149 (13.4)**	**<0.001** *
**History of ADRs, *n* (%)**	**439 (28.2)**	**156 (35.1)**	**283 (25.4)**	**<0.001** *

*Note*: The “history of ADRs” item included ADRs that occurred before the episode of AKI and were mentioned in the patient's EMRs. Quantitative variables are quoted as the mean (standard deviation), and qualitative variables are quoted as the frequency (percentage).

Abbreviations: ADR, adverse drug reaction; AKI, acute kidney injury; BMI, body mass index; CKD, chronic kidney disease; CVD, cardiovascular disease. Statistically significant differences are shown in bold type.

^a^ In Wilcoxon's test or a chi‐squared test, for patients with drug‐induced AKI versus patients with non‐drug‐induced AKI.

^b^ According to the patients' medical records.

* Statistically significant.

### 3.2 Characteristics of drug‐induced AKIs

Of the 1557 AKIs, 445 (28.6%) were drug‐induced (Table 1). The mean (SD) age of patients with a drug‐induced AKI was 72.1 (14.6). The patients' characteristics are summarized in Table 1. Relative to patients with a non‐drug‐induced AKI, patients with a drug‐induced AKI were significantly more likely to be women (43.6% vs. 51.0%, respectively), to be obese (6.6% vs. 6.9%), and to have comorbidities. Drug‐induced AKIs were significantly more likely to have a favorable outcome (in 69.9% of patients vs. 65.7% of patients with non‐drug‐induced AKI) but were also more likely to lead to the onset or progression of CKD (19.8% vs. 13.7%, respectively). Drug‐induced AKIs were more likely to be managed in the nephrology department, whereas non‐drug‐induced AKIs were more likely to be managed in the ICU (*P* < 0.001) (Table 2).

**TABLE 2:** Characteristics of the AKIs, overall and by group (drug‐induced or non‐drug‐induced)

	All patients with AKI	Drug‐induced AKI	Non‐drug‐induced AKI	*P* value^a^
	1557	445 (28.6)	1112 (71.4)	
Hospital‐acquired	924 (59.3)	265 (59.6)	659 (59.3)	0.92
**Medical unit**				**<0.001** *
**Nephrology, *n* (%)**	**151 (9.3)**	**65 (14.6)**	**86 (7.7)**	
**ICU, *n* (%)**	**409 (26.0)**	**59 (13.3)**	**350 (31.5)**	
**Other, *n* (%)**	**997 (64.7)**	**321 (72.1)**	**676 (60.8)**	
**Length of hospital stay, mean (SD)**	**17.6 (19.8)**	**16.0 (16.3)**	**18.2 (21.0)**	**<0.001** *
Type of stay, *n* (%)				0.09
Short (<7 days)	400 (25.7)	123 (27.6)	277 (24.9)	
Medium (7–29 days)	913 (58.6)	266 (59.8)	647 (58.2)	
Long (>29 days)	244 (15.7)	56 (12.6)	188 (16.9)	
KDIGO grade				0.13
1 (*n*, %)	843 (54.2)	259 (58.2)	584 (52.5)	
2 (*n*, %)	371 (23.8)	96 (21.6)	275 (24.7)	
3 (*n*, %)	343 (22.0)	90 (20.2)	253 (22.8)	
Use of dialysis, *n* (%)	146 (9.4)	38 (8.5)	108 (9.7)	0.47
**Outcome**				**<0.001** *
**Favorable, *n* (%)**	**1042 (67.0)**	**311 (69.9)**	**731 (65.7)**	
**Onset or progression of CKD, *n* (%)**	**240 (15.4)**	**88 (19.8)**	**152 (13.7)**	
**Death, *n* (%)**	**233 (15.0)**	**35 (7.9)**	**198 (17.8)**	
**Not known, *n* (%)**	**42 (2.6)**	**11 (2.5)**	**31 (2.8)**	

*Note*: Quantitative variables are quoted as the mean (standard deviation), and qualitative variables are quoted as the frequency (percentage).

Abbreviations: AKI, acute kidney injury; CKD, chronic kidney disease; ICU, intensive care unit; KDIGO, Kidney Disease: Improving Global Outcomes. Statistically significant differences are shown in bold type.

^a^ In a Wilcoxon test or a chi‐squared test, for patients with drug‐induced AKI versus patients with non‐drug‐induced AKI.

* Statistically significant.

In drug‐induced AKI, the mean (SD) number of involved drugs was 2.3 (1.7). A total of 1009 drugs were imputed with Naranjo scale (464 for CA‐AKIs and 545 for HA‐AKIs). The main ATC classes involved were diuretics (32.2%), renin‐angiotensin drugs (17.3%), and antibiotics (7.8%) (Table 3). The main imputed antibiotic classes were penicillins (2.5%), sulfamides (1.3%), and fluoroquinolones (1.1%) (Table S2). The main specifically imputed drugs were furosemide (21.8%), spironolactone (5.8%), and ramipril (5.1%). For more details, see Table S3.

**TABLE 3:** Main drug classifications involved in drug‐induced AKI

	All drug‐induced AKIs (*n*)	CA drug‐induced AKI (*n*, %)	HA‐drug‐induced AKI (*n*, %)	*P* value
	*N* = 445	*N* = 180 (40.4)	*N* = 265 (59.6)	
**Number of drugs (mean, SD)**	**2.3 (1.7)**	**2.6 (1.7)**	**3.0 (1.5)**	**<0.001** *
**ATC (n, %)**				
**Diuretics**	**325 (32.2)**	**119 (25.6)**	**206 (37.8)**	**<0.001** *
Renin‐angiotensin drugs	175 (17.3)	87 (18.8)	88 (16.1)	0.19
**Antibacterial agents for systemic use**	**79 (7.8)**	**12 (2.6)**	**67 (12.3)**	**<0.001** *
**Antineoplastic agents**	**57 (5.6)**	**37 (8.0)**	**20 (3.7)**	**<0.001** *
Antithrombotics	53 (5.3)	29 (6.3)	24 (4.4)	0.19
**Antidiabetics, except insulin**	**46 (4.6)**	**37 (8.0)**	**9 (1.7)**	**<0.001** *
Psycholeptics	34 (3.4)	21 (4.5)	13 (2.4)	0.06
**Contrast agents**	**28 (2.8)**	**3 (0.6)**	**25 (4.6)**	**<0.001** *
Analgesics	27 (2.7)	13 (2.8)	14 (2.6)	0.82
**Immunosuppressive agents**	**25 (2.5)**	**18 (3.9)**	**7 (1.3)**	**<0.001** *
Beta‐blockers	20 (2.0)	7 (1.5)	13 (2.4)	0.32
**Lipid‐lowering agents**	**15 (1.5)**	**12 (2.6)**	**3 (0.6)**	<0.001**
NSAIDs	13 (1.3)	9 (1.9)	4 (0.7)	0.10
Calcium channel blockers	13 (1.3)	9 (1.9)	4 (0.7)	0.10
Anti‐gout drugs	11 (1.1)	7 (1.5)	4 (0.7)	0.36

*Note*: Quantitative variables are quoted as the mean (standard deviation), and qualitative variables are quoted as the frequency (percentage).

Abbreviations: AKI, acute kidney injury; CA, community‐acquired; HA, hospital‐acquired, NSAID, non‐steroidal anti‐inflammatory drug; SD, standard deviation. Statistically significant differences are shown in bold type.

* Statistically significant.

An adjusted multivariate logistic regression identified several significant risk factors for drug‐induced AKI: female sex (OR [95% CI] = 1.3 [1.04–1.67]), CKD (1.8 [1.40–2.67]), and a previous ADR (1.3 [1.05–1.73]) (Table 4).

**TABLE 4:** The regression model for the risk of drug‐induced AKI (*N* = 445)

Predictors	Crude OR [95% CI]	*P* value	Adjusted OR [95% CI]^a^	Adjusted *P* value^a^
**Demographics**				
Age, y, mean (SD)	1.0 [0.99–1.01]	0.23	0.99 [0.98–1.01]	0.57
**Sex**				
**Male**	**Reference**		**Reference**	
**Female, *n* (%)**	**1.3 [1.01–1.68]**	**<0.001** *	**1.3 [1.04–1.67]**	**0.016** *
Obesity				
BMI < 30 kg/m^2^	Reference		Reference	
BMI ≥ 30 kg/m^2^, *n* (%)	1.4 [1.08–1.73]	9.05 e‐3*	1.2|0.92–1.52]	0.20
**Comorbidities** ^b^				
**CKD**				
**No history of CKD**	**Reference**		**Reference**	
**History of CKD**	**2.2 [1.72–2.74]**	**<0.001** *	**1.8 [1.40–2.67]**	**<0.001** *
Hypertension				
No hypertension	Reference		Reference	
Hypertension	1.6 [1.21–2.00]	<0.001*	1.2 [0.87–1.55]	0.31
Diabetes				
No diabetes	Reference		Reference	
Diabetes	1.3 [1.05–1.66]	1.58 e‐2*	0.97 [0.75–1.25]	0.81
Dyslipidaemia				
No dyslipidaemia	Reference		Reference	
Dyslipidaemia	1.5 [1.21–1.90]	<0.001*	1.3 [0.99–1.63]	0.06
History of CVD				
No history of CVD	Reference		Reference	
History of CVD	1.5 [1.22–1.91]	<0.001*	1.3 [0.99–1.61]	0.07
History of gout				
No history of gout	Reference		Reference	
History of gout	1.4 [0.90–2.04]	0.13	1.0 [0.67–1.57]	0.87
Kidney transplantation				
No history of kidney transplantation	Reference		Reference	
History of kidney transplantation	1.8 [0.97–3.12]	0.05	1.0 [0.55–1.94]	0.90
History of AKI				
No history of AKI	Reference		Reference	
History of AKI	1.7 [1.28–2.27]	<0.001*	1.2 [0.87–1.62]	0.27
**History of ADRs**				
**No history of ADRs**	**Reference**		**Reference**	
**History of ADRs**	**1.6 [1.25–2.00]**	**<0.001** *	**1.3 [1.05–1.73]**	**<0.001** *

*Note*: Statistically significant differences are shown in bold type.

Abbreviations: ADRs, adverse drug reactions; AKI, acute kidney injury; BMI, body mass index; CI, confidence interval; CKD, chronic kidney disease; CVD, cardiovascular disease; OR, odds ratio.

^a^ Adjusted for variables with *P* < 0.2 in a univariate analysis or contextual interest.

^b^ According to the patients' EMRs.

* Statistically significant.

### 3.3 Comparison of HA‐AKIs and CA‐AKIs

Of the 445 drug‐induced AKIs, 180 (40.4%) were classified as CA‐AKIs, and 265 (59.6%) were classified as HA‐AKIs (Table 3). In both subgroups, the most frequently involved drugs were diuretics (25.6% for CA‐AKI and 37.8% for HA‐AKI) and renin‐angiotensin drugs (18.8% for CA‐AKI and 16.1% for HA‐AKI). For more details, see Table 3.

Antibiotic, diuretics, and contrast agents were significantly more likely to be involved in HA‐AKI, whereas antineoplastic, lipid‐lowering drugs, antidiabetics, and immunosuppressive agents were significantly more likely to be involved in CA‐AKI (Table 3).

CKD was a significant risk factor in both subgroups (OR [95% CI] = 1.9 [1.22–2.82]) for CA‐AKI and 1.8 [1.29–2.58] for HA‐AKI). Female sex was a risk factor for CA‐AKI only (Tables 5 and 6).

**TABLE 5:** Regression model for risk of HA drug‐induced AKI (*n* = 265)

Predictors	Crude OR [95% CI]	*P* value	Adjusted OR [95% CI]^a^	Adjusted *P* value
**Demographics**				
Age, y, mean (SD)	1.0 [1.00–1.02]	0.04*	1.0 [80.99–1.01]	0.79
Sex				
Men	Reference		Reference	
Women, *n* (%)	1.3 [0.99–1.76]	0.06	1.3 [0.94–1.74]	0.11
Obesity				
BMI < 30 kg/m^2^	Reference		Reference	
BMI ≥ 30 kg/m^2^, *n* (%)	1.3 [0.94–1.72]	0.12	1.1 [0.78–1.51]	0.60
**Comorbidities** ^b^				
**CKD**				
**No history of CKD**	**Reference**		**Reference**	
**History of CKD**	**2.2 [1.61–3.01]**	**<0.001** *	**1.8 [1.29–2.58]**	**<0.001** *
Hypertension				
No hypertension	Reference		Reference	
Hypertension	1.6|1.13–2.18]	7.02 e‐3*	1.9 [0.75–1.59]	0.66
Diabetes				
No diabetes	Reference		Reference	
Diabetes	1.3 [0.96–1.75]	0.09	0.9 [0.66–1.31]	0.69
**Dyslipidaemia**				
**No dyslipidaemia**	**Reference**		**Reference**	
**Dyslipidaemia**	**1.9 [1.41–2.52]**	**<0.001** *	**1.6 [1.20–2.28]**	**<0.001** *
History of CVD				
No history of CVD	Reference		Reference	
History of CVD	1.7 [1.25–2.24]	<0.001*	1.3 [0.92–1.76]	0.14
Cancer				
No cancer	Reference		Reference	
With cancer	0.8 [0.58–1.10]	0.18	0.9 [0.61–1.19]	0.36
Kidney transplantation				
No history of kidney transplantation	Reference		Reference	
History of kidney transplantation	2.0 [0.69–5.31]	0.18	1.1 [0.38–3.30]	0.80
History of AKI				
No history of AKI	Reference		Reference	
History of AKI	1.6 [1.08–2.32]	1.65 e‐2*	1.1 [0.74–1.68]	0.59
History of ADRs				
No history of ADRs	Reference		Reference	
History of ADRs	1.4 [1.06–1.99]	1.92 e‐2*	1.3 [0.93–1.80]	0.12

*Note*: Statistically significant differences are shown in bold type.

Abbreviations: ADRs, adverse drug reactions; AKI, acute kidney injury; BMI, body mass index; CI, confidence interval; CKD, chronic kidney disease; CVD, cardiovascular disease; OR, odds ratio.

^a^ Adjusted for variables with *P* < 0.2 in a univariate analysis or contextual interest.

^b^ According to the patients' EMRs.

* Statistically significant.

**TABLE 6:** The regression model for the risk of CA drug‐induced AKI (*N* = 180)

	Crude OR [95% CI]	*P* value	Adjusted OR [95% CI]^a^	Adjusted *P* value
**Demographics**				
Age, y, mean (SD)	0.99 [0.98–1.01]	0.57	0.99 [0.98–1.00]	0.17
**Sex**				
**Male**	**Reference**		**Reference**	
**Female, *n* (%)**	**1.4 [0.98–1.96]**	**0.07**	**1.5 [1.02–2.14]**	**0.04** *
Obesity				
BMI < 30 kg/m^2^	Reference		Reference	
BMI ≥ 30 kg/m^2^, *n* (%)	1.5 [1.05–2.25]	2.51 e‐2*	1.3 [0.89–2.02]	0.16
**Comorbidities** ^b^				
**CKD**				
**No history of CKD**	**Reference**		**Reference**	
**History of CKD**	**2.2 [1.52–3.11]**	**<0.001** *	**1.9 [1.22–2.82]**	**<0.001** *
Hypertension				
No hypertension	Reference		Reference	
Hypertension	1.5 [1.04–2.30]	3.52 e‐2*	1.2 [0.74–1.88]	0.50
Diabetes				
No diabetes	Reference		Reference	
With diabetes	1.4 [0.96–1.93]	0.09	1.0 [0.71–1.54]	0.82
History of CVD				
No history of CVD	Reference		Reference	
History of CVD	1.4 [0.96–1.93]	0.08	1.3 [0.86–1.89]	0.23
**Cancer**				
**No cancer**	**Reference**		**Reference**	
**Cancer**	**1.7 [1.15–2.40]**	**6.35 e‐3** *	**1.9 [1.29–2.79]**	**<0.001** *
History of gout				
No history of gout	Reference		Reference	
History of gout	1.7 [0.93–3.05]	0.08	1.4 [0.75–2.67]	0.27
Kidney transplantation				
No history of kidney transplantation	Reference		Reference	
History of kidney transplantation	1.7 [0.80–3.43]	0.15	1.0 [0.45–2.24]	0.96
History of AKI				
No history of AKI	Reference		Reference	
History of AKI	1.9 [1.21–2.91]	4.03 e‐3*	1.2 [0.74–1.97]	0.43
History of ADRs				
No history of ADRs	Reference		Reference	
History of ADRs	1.8 [1.24–2.57]	1.75 e‐3*	1.4 [0.91–2.05]	0.12

*Note*: Statistically significant differences are shown in bold type.

Abbreviations: ADRs, adverse drug reactions; AKI, acute kidney injury; BMI, body mass index; CI, confidence interval; CKD, chronic kidney disease; CVD, cardiovascular disease; OR, odds ratio.

^a^ Adjusted for variables with *P* < 0.2 in a univariate analysis or contextual interest.

^b^ According to the patients' EMRs.

* Statistically significant.

## 4 DISCUSSION

Our study of hospitalized patients with AKI highlighted the high proportion (28.6%) of patients with drug‐induced AKI. Our comprehensive analysis of determinants of drug‐induced AKI enabled us to identify female sex, CKD, and a history of ADRs as risk factors in this population. The comparison between CA‐AKI and HA‐AKI drug‐induced highlights that patient characteristics, drugs, and the issues are not the same and that the key messages are different. Indeed, general practitioners and clinicians should be more cautious when prescribing these drugs—especially in at‐risk patients such as those with CKD.

The proportion of drug‐induced AKIs in our cohort (28.6%) was slightly higher than in the epidemiological literature (between 14.4% and 20.0%, depending on the study [11, 25, 26]). This disparity with the literature data might be to the relatively advanced age of our study population (mean age: 71.3). Age is a well known risk factor for AKI [27]: The risk of developing AKI is 3.5 times greater in patients aged over 65 or 70 than in younger patients [28, 29]. Moreover, AKIs often have several causes and are aggravated by CKD, cardiovascular morbidities, and polypharmacy. Robert et al. showed that 63.2% of community‐acquired drug‐induced AKIs had several contributory factors [8].

The main drugs involved in AKIs in our study were similar to those mentioned in the literature. It is well known that diuretics and angiotensin‐converting enzyme inhibitors are frequently involved in AKI [10]. By decreasing the synthesis of angiotensin II, angiotensin‐converting enzyme inhibitors alter intraglomerular haemodynamics and thus decrease the GFR. This hemodynamic disorder may result in nephrotoxicity—especially in cases of reduced renal blood flow [15, 17]. The role of loop diuretics in AKI is subject to debate. These drugs are prescribed frequently in emergency and intensive care medicine for the treatment of congestive heart failure, since they enable the elimination of large amounts of electrolytes and water if the kidneys are still able to filter. As also reported in the literature, the most frequently prescribed drug in our study was furosemide. However, AKIs with various aetiologies are characterized by a low GFR [30]. According to a multicentre prospective study conducted in Shanghai, diuretics were responsible for 22.2% of all drug‐induced AKIs [31]. Diuretics may reduce glomerular filtration—leading to functional AKI—and can also induce acute interstitial nephritis via an immunoallergic mechanism [15].

Using the Naranjo scale for ADRs, we found that CA‐AKIs and HA‐AKIs involved different ATC classes. Antibiotic, diuretics, and contrast agents were significantly more likely to be involved in HA‐AKI, which is in line with the literature data [21]. This might be due to the AKI's etiology: Radiological examinations and the management of sepsis and CVD occur in hospital and require the administration of these types of drug [32]. Antibiotics can induce AKI through several mechanisms [15].

Contrast agents have a direct nephrotoxic effect on tubular epithelial cells, with the release of vasoactive molecules, an increase in oxidative stress, and subsequent ischaemic renal cell injury [33].

Antineoplastic, antidiabetic, and immunosuppressive agents were significantly more frequently involved in CA‐AKI. This might have been due to the characteristics of the affected population, such as a greater comorbidity burden and more frequent polypharmacy for chronic health conditions. Antineoplastic agents can produce chronic interstitial injury by inducing oxidative stress, apoptosis, necrosis, local and systemic inflammation, the release of inflammatory mediators, and autophagy [34]. Immunosuppressive drugs may induce acute tubular necrosis, chronic interstitial nephritis, and thrombotic microangiopathy [15].

Drug‐induced AKIs are often avoidable [8, 35]. Given that different types of medication are involved in CA‐AKI and HA‐AKI, it might be possible to adjust and improve the management of these injuries as a function of the affected population. In hospital, the dose level of diuretics (like furosemide) should be adjusted according to the individual patient's diuresis rate and comorbidities [36]. Various ways of preventing contrast‐induced AKI have been documented, and the guidelines have been updated [37]: increase hydration in patients with CKD, initiate prophylaxis with intravenous saline and intravenous sodium bicarbonate, and withdraw nephrotoxic drugs before contrast agent are administered. The use of medication to reduce the oxidative stress induced by contrast agents has also been studied [38].

In the community, optimal hydration should be constantly encouraged and monitored in patients at risk of drug‐induced AKI. This approach encompasses patient education and prescription policies. Combinations of nephrotoxic drugs should be avoided whenever possible and especially in patients with polypharmacy [11]. The use of over‐the‐counter medications should be checked and monitored, depending on the patient's comorbidities (e.g., CKD) and prescription medications.

Our multivariate analysis identified several significant risk factors for drug‐induced AKI: female sex, CKD, and previous ADR. These findings were consistent with the literature data [11]. A sex difference in ADRs has been well documented [39]; Moyer et al. found a 1.6‐fold‐greater OR in women than in men, for example [40]. Historically, women were less likely than men to be included in clinical trials. Moreover, it has been reported that specific physiological features of women (i.e., hormonal status) can alter the pharmacokinetics and pharmacodynamics of medications [40], and this influences the response to injury and/or treatment [41]. Indeed, Watson et al.'s post‐marketing surveillance analysis of spontaneous reports in VigiBase (the World Health Organization's global database of individual case safety reports) found that women (and especially those of child‐bearing age) reported more ADRs than men did [42].

Several studies have found that a history of ADRs is a risk factor for further ADRs [35, 43]. This might be due to immune memory reactions (such as antibiotic‐induced AKIs [44, 45, 46]) that tend to become more severe upon repeated exposure. Another explanation might relate to the characteristics of the patients with ADRs: These individuals tended to be frailer, with more comorbidities (particularly pre‐existing renal disease, preceded by polypharmacy) [35]. However, most studies have focused on ADRs in general and not specifically on drug‐induced AKI. Our present results found that a history of ADRs was a risk factor for drug‐induced AKI. This shows why the physician should know the details of the patient's medical and medication history when seeking to prevent drug‐induced AKI.

A history of CKD was a consistent risk factor for drug‐induced AKI overall and for the CA‐AKI and HA‐AKI subgroups. The relationship between CKD and AKI has already been highlighted by several studies [2, 3]. Firstly, CKD is known to predispose or sensitize patients to AKI and to slow down the kidneys' ability to recover [47]; in a large cohort of patients with CKD, we highlighted the high prevalence of ADRs when drug‐induced AKI had been the first reported serious ADR [48]. Secondly, AKI may contribute to the development and progression of CKD through the failure of self‐regulation, abnormal vasodilation, sensitivity to antihypertensive drugs, and the occurrence of ADRs [47]. Moreover, patients with a low GFR have an elevated risk of ADRs, due to impairments in drug metabolism and excretion and the greater accumulation of parent drugs and their metabolites [49]. Surprisingly, a history of AKI was not found to be a risk factor for drug‐induced AKI in our multivariate analysis—even though AKI's harmful effects on kidney recovery are well described [2, 3]. Likewise, history of AKI was not a risk factor for CA‐AKI or HA‐AKI.

In the literature, drug‐induced AKIs are often compared with non‐drug‐induced AKIs. Few studies have described the influence of drugs on AKI outcomes. Alkhunaizi et al. showed that patients with drug‐induced AKI were younger, more likely to be female and more likely to have CKD than those with non‐drug‐induced AKI but did not report on the outcomes [50]. Here, we compared outcomes in drug‐induced AKI versus non‐drug‐induced AKI. Drug‐induced AKIs were more likely to have a favorable outcome but were also more likely to lead to the onset or progression of CKD. Drug‐induced AKIs were more likely to be managed in a nephrology department. This might be due to the higher prevalence of CKD in the drug‐induced AKI group (40.4%) than in the non‐drug‐induced AKI group (23.8%). Furthermore, the majority of AKIs analyzed here were not managed in a nephrology department—probably because AKI is often multifactorial and is associated with other pathologies, such as sepsis and heart failure.

The length of stay was shorter for drug‐induced AKIs than for non‐drug‐induced AKIs. A deeper understanding of these data might help to improve the management of drug‐induced AKI and to preserve kidney function.

Our study had a number of strengths. Firstly, the AKI cases were selected rigorously by applying the KDIGO guidelines (an international benchmark) and by screening the EMRs. Secondly, all ADRs were fully reviewed by a pharmacovigilance specialist. Thirdly, the consecutive patient recruitment and the large numbers of included AKIs enabled us to describe drug‐induced events and evaluate them according to where they occurred (i.e., in hospital or in the community). To the best of our knowledge, the present study is the first to have determined the proportions of HA‐AKI and CA‐AKIs in a cohort of patients with drug‐induced AKIs and to have determined the affected patients' characteristics and outcomes.

Our study had some limitations. Firstly, our single‐center study was conducted in a university hospital in northern France, and so our findings might reflect the prescribing habits of our local general practitioners and the hospital's clinicians. However, as shown by the literature data, we expect the clinical characteristics of patients with drug‐induced AKI to be much the same in France as in other countries. Secondly, we focused on a specific cause of AKI, even though the latter it is often multifactorial. Hence, it would be useful to determine whether certain causes are more likely than others to be associated with poorer outcomes. Thirdly, we did not collect information on the use of over‐the‐counter drugs or self‐medication; this might have led us to underestimate exposure to certain drugs such as NSAIDs. Indeed, analgesics like NSAIDs are frequently used for self‐medication, and the continuous use of NSAID (alone or combined with other drugs) for pain relief over long periods of time is associated with the development of progressive kidney disease or AKI [51]. Information on self‐medication rarely recorded in hospital discharge summary databases like the PMSI [52] and is not systematically recorded in EMRs. Furthermore, AKI cases were evaluated by a single adjudicator; a manually review of all EMRs was a time‐consuming process that prevented a second evaluation. We did not have access to urine output, which could have led to an underestimation of AKI cases. However, our algorithm is known to be highly specific for AKI [7], and our aim was not to calculate the prevalence of AKI. Lastly, patients were not followed up after discharge from hospital (preventing us from evaluating the long‐term outcomes associated with drug‐induced AKI), and our study period was short (6 months). Despite these limitations, our large, comparative study of CA‐AKI and HA‐AKI provided a better understanding of the epidemiology, risk factors, course, and outcomes for these two specific types of drug‐induced AKI.

## 5 CONCLUSION

Drug‐induced AKIs account for a large (and possible underestimated) proportion of community‐ and hospital‐acquired AKIs. The serious consequences of AKI on kidney and the potential for further ADRs mean that efforts should be made to optimize treatment and understanding all the risk factors in specific populations (such as those with community‐ or hospital‐acquired AKIs). Indeed, we found some differences between the CA‐AKI and HA‐AKI subgroups with regard to patient characteristics and outcomes. Since a large proportion of drug‐induced AKIs are avoidable, the close monitoring of female patients with multiple comorbidities (particularly CKD and a history of ADRs) might limit the risk of drug‐induced AKI and thus have a positive impact on healthcare costs and patient care. To study AKI in more depth and improve the quality of the data, future research might usefully include a longer follow‐up period, a larger, multicentre cohort, the use of natural language processing (to avoid time‐consuming reviews), and a pharmacovigilance database.

## CONFLICT OF INTEREST

The authors declare that they have no competing interests.

## ETHICS APPROVAL

The IRA‐PMSI study's objectives and procedures were approved by an independent ethics committee (*CPP Nord Ouest II*, Amiens, France; reference: RCB 2020‐A00556‐33).

## PATIENT CONSENT

In line with the French legislation on non‐interventional studies, patients were provided with information about the study and were free to refuse to participate.

## CLINICAL TRIAL REGISTRATION

The study was registered at ClinicalTrials.gov (NCT04923750).

## AUTHOR CONTRIBUTIONS

AR was a major contributor to the acquisition, analysis, and interpretation of data; drafted the article; and revised it critically for important intellectual content. GC and KM were the major contributors to analysis and interpretation of the data and revised the article critically for important intellectual content. VG and SL were the major contributors to the study conception and design and acquisition, analysis, and interpretation of the data; drafted the article, and revised it critically for important intellectual content. All authors read and approved the final manuscript.

## Supporting information


**Data S1.** Supplementary.T1: Distribution of the cohort sources
Supplementary.T2: Imputed antibacterial classes for drug‐induced AKIs
Supplementary.T3: Main imputed drugs for drug‐induced AKIs in the overall study population

## Data Availability

The datasets used and/or analyzed during the current study are available from the corresponding author on reasonable request.
