# Supplementary material for: Risk factors for and characteristics of community‐ and hospital‐acquired drug‐induced acute kidney injuries
Source: Fundam Clin Pharmacol. 2022 Jan 25;36(4):750–61. doi: 10.1111/fcp.12758 (PMC9545588; doi:10.1111/fcp.12758)
Supplement: Supplementary file 1 — Data S1. Supplementary.T1: Distribution of the cohort sources Supplementary.T2: Imputed antibacterial classes for drug‐induced AKIs Supplementary.T3: Main imputed drugs for drug‐induced AKIs in the overall study population [file FCP-36-750-s001.docx]

**Risk factors for and characteristics of community- and hospital-acquired drug-induced acute kidney injuries**

Amayelle **Rey**, PharmD ^1,2^, Valérie **Gras-Champel**, PharmD, PhD^1,2^, Gabriel **Choukroun**, MD, PhD ^2,3^, Kamel **Masmoudi**, MD^1^, Sophie **Liabeuf**, PharmD, PhD^1,2,*^

^1^ Division of Clinical Pharmacology, Amiens University Hospital, Amiens, France

^2^ MP3CV Laboratory, EA7517, Jules Verne University of Picardie, Amiens, France

^3^ Division of Nephrology, Amiens University Hospital, Amiens, France

***Corresponding author**: Sophie Liabeuf, Division of Clinical Pharmacology, Amiens University Hospital, Avenue René Laennec, F-80000 Amiens, France.

Tel.: +33-322-087-092; Fax: +33-322-087-095; E-mail: rey.amayelle@chu-amiens.fr

**Supplementary method**

*Study design and population*

The *Programme de médicalisation des systèmes d’information* (PMSI) is the French national hospital discharge summary database. It notably includes the hospital identifier, hospital stay start and end dates, and the diagnostic codes (a primary diagnosis, a related diagnosis, and up to 30 associated diagnoses, coded according to the International Classification of Diseases, 10^th^ Revision (ICD-10)) [1]. We performed a single-centre, retrospective study (“IRA-PMSI”) from January 1^st^, 2019, to June 30^st^, 2019, in Amiens Picardie University Hospital (Amiens, France).

The main inclusion criteria were age over 18 years and at least two available plasma creatinine measurements during the hospital stay, with a 1.5-fold increase or more. We excluded patients with a diagnosis of end stage renal disease (ESRD) requiring dialysis, kidney transplantation during the study period and postpartum AKI. This information was manually looked for in electronic medical records (EMR). Patients admitted through the hospital’s emergency departments, in a long-term care unit and for laboratory tests only were also excluded because the ICD-10 codes for their stays were not available in the PMSI, and these data were necessary for our primary objectives which have already been published [2].

*Definition of cases of AKI*

All EMRs for hospitalized patients with AKI were manually reviewed and screened against the study’s inclusion and exclusion criteria, as follows. Only one AKI per hospital stay was studied. If a patient had experienced several AKIs during a single hospital stay, only the first AKI to occur was selected for analysis. If a patient had more one hospital stay with an AKI, the stays were considered independently; the previous AKI was recorded as an history of AKI for the following episode. Indeed, the patient’s comorbidities and the nature of the AKI tended to differ from one hospital stay to another

We built an algorithm to screen the EMRs and laboratory data for cases of CA-AKI and HA-AKI based on physician-diagnosed for AKI and then for an elevated plasma creatinine level (**supplementary** **Figure 1**). This approach ensured that we would not miss an AKI episode when a baseline creatinine value was missing (after measurement in a clinical laboratory outside hospital, for example), and is known to be highly specific for AKI [2]. For HA-AKI, the peak creatinine level measured during a hospital stay was used to define the presence or absence of AKI. If several baseline creatinine values were available, the most recent value was used. For CA-AKI, we accessed the patient’s laboratory results: If creatinine measurements were available in the 7 days prior to hospital admission, they were considered to be the baseline measurements. If not, we looked for a creatinine baseline measurement in the previous EMRs (**supplementary** **Figure 1**).

Urine output was not used as a criterion for AKI, since this variable was not available in the study database.

Plasma creatinine levels were measured using an Atellica*®* CH analyzer (Siemens Healthineers).

Bibliography

[1] Boudemaghe T., Belhadj I. Data Resource Profile: The French National Uniform Hospital Discharge Data Set Database (PMSI). *International Journal of Epidemiology*. 2017;46(2):392-392d.

[2] Rey A., Gras-Champel V., Balcaen T., et al. Use of a hospital administrative database to identify and characterize community-acquired, hospital-acquired and drug-induced acute kidney injury. *J Nephrol*. Published online October 7, 2021.

**Supplementary Figure 1: Algorithm for identifying cases of CA-AKI or HA-AKI**

AKI: acute kidney injury; CA: community-acquired; CKD: chronic kidney disease; HA: hospital-acquired; EMR: electronic medical record.

**Supplementary materials**

**Supplementary.T1: Distribution of the cohort sources**

| **Type of review** | **Definition** | **N (%)** |
| --- | --- | --- |
| **Laboratory values** |  |  |
| AKI_cr_ | AKI identified from the creatinine values, according to the KDIGO definition only | 188 (12.1) |
| **EMRs** |  |  |
| AKI_EMR_ | AKI identified by a physician from the EMRs only | 515 (33.1) |
| **Both reviews** |  |  |
| AKI | AKI identified from the creatinine values and the EMR | 854 (54.8) |

**Supplementary.T2: Imputed antibacterial classes for drug-induced AKIs**

| **Class** | **All drugs**  **N=1009** | **Drugs in CA-AKI**  **N= 464** | **Drugs in HA-AKI**  **N= 545** |
| --- | --- | --- | --- |
| Penicillin | 25 (2.5) | 3 (0.6) | 22 (4.0) |
| Sulfamide antibacterials | 13 (1.3) | 4 (0.9) | 9 (1.7) |
| Fluoroquinolone | 11 (1.1) | 1 (0.2) | 10 (1.8) |
| Aminoglycoside | 10 (1.0) | 1 (0.2) | 9 (1.7) |
| Cephalosporin | 6 (0.6) | 0 (0) | 6 (1.1) |
| Glycopeptide | 5 (0.5) | 0 (0) | 5 (0.9) |
| Oxazolidinone | 4 (0.4) | 1 (0.2) | 3 (0.6) |
| Rifamycin | 2 (0.2) | 0 (0) | 2 (0.4) |
| Lincosamide | 1 (0.1) | 0 (0) | 1 (0.2) |
| Macrolide | 1 (0.1) | 1 (0.2) | 0 (0) |
| Tetracycline | 1 (0.1) | 1 (0.2) | 0 (0) |

**Supplementary.T3: Main imputed drugs for drug-induced AKIs in the overall study population**

| **Drugs** | **N** | **%** |
| --- | --- | --- |
|  | **1009** |  |
| Furosemide | 220 | 21.8 |
| Spironolactone | 58 | 5.8 |
| Ramipril | 51 | 5.1 |
| Metformin | 33 | 3.3 |
| Perindopril | 31 | 3.1 |
| Hydrochlorothiazide | 30 | 3.0 |
| Candesartan | 29 | 2.9 |
| Contrast agents | 28 | 2.8 |
| Irbesartan | 18 | 1.8 |
| Valsartan | 14 | 1.4 |
| Trimethoprim-sulfamethoxazole | 13 | 1.3 |
